# Outcomes from arts‐based engagements delivered in green spaces for people living with neurological conditions: A scoping review

**DOI:** 10.1002/alz70860_099600

**Published:** 2025-12-23

**Authors:** Funmi Akindejoye, Azam Bazooband, Duncan Sinclair, Pauline Marsh

**Affiliations:** ^1^ University of Tasmania, Tasmania, TAS, Australia; ^2^ UNIVERSITY OF TASMANIA, HOBART, TAS, Australia; ^3^ University of Tasmania, Hobart, TAS, Australia

## Abstract

**Background:**

As rates of neurological conditions rise, a pressing global public health challenge is emerging. While pharmacological prescriptions can slow some disease advancement, they currently do not offer a cure. Non‐pharmacological interventions, however, have shown promise in improving the quality of life and psychosocial aspects of living with various neurological conditions. Two such interventions ‐ engaging with nature and participating in creative arts ‐ have proven to be beneficial. This scoping review aims to identify the types and delivery mode of art‐ engagements in green spaces for people living with neurological conditions and explore the empirical outcomes of participating in such art activities.

**Method:**

A systematic search for published evidence was conducted between April to June 2024. We included studies using the following criteria: non‐pharmacological interventions including art engagement that were conducted in green spaces involving people living with neurological conditions.

**Result:**

Five articles met the inclusion criteria. Participants were living with Alzheimer's disease and other types of dementia, and late‐stage Huntington's disease. Art engagements ranged from display of personal artworks, theatre performance, community quilt making, sculptural art installation, craft making with garden products, sculptural mosaic and painting. The green spaces where these engagements were performed included outdoor healing gardens, indoor gardens and historical parks. Outcomes included positive emotions, enhanced social connection, feelings of achievement and meaning, enhanced reminisce, improved wellbeing, enhanced sensory stimulation and increased connection to nature.

**Conclusion:**

Beyond the documented beneficial outcomes of art engagements in green spaces, the combination of arts and the nature setting provided more opportunities for cultural inclusivity, diversity in delivery of art engagements, meaningful activities in nature, enhanced nature connection and encouraged intergenerational participation. The combination of art and nature provided opportunities for cultural inclusivity, diversity in delivery of art engagements, meaningful activities in nature, enhanced nature connection and encouraged intergenerational participation. Also, group participation and multidisciplinary collaboration were common features across reviewed art engagements in nature. There is need for future related research obtain direct data from people with neurological conditions, and explore facilitators and barriers of art engagements within green spaces.

